# Multi-jurisdictional Prostate Cancer Registry Linkage.

**DOI:** 10.23889/ijpds.v7i3.2101

**Published:** 2022-08-25

**Authors:** Christopher Radbone, Kerri Beckmann, David Roder, Michael O'Callaghan, Kim Moretti, Liesel Fitzgerald, Liz Buckley

**Affiliations:** 1 SA NT DataLink; 2 University of South Australia; 3 SA Health; 4 South Australian Prostate Cancer Clinical Outcome Collaborative (SA-PCCOC); 5 University of Tasmania; 6 Flinders University

## Objectives

The Prostate Cancer Outcomes Registry for Australian and New Zealand (PCOR-ANZ) aims to monitor population-wide prostate cancer characteristics, treatments, and outcomes. However, long-term follow-up of secondary treatments, disease progression, and side effects is limited. This novel project provides increased utility, enhancing this clinical registry by integrating national and jurisdictional data.

## Approach

The Tasmanian and South Australian jurisdictional registries (PCOR-Tas and PCOR-SA) are being treated as pilots for this nationally relevant data linkage project. Each contains descriptive data on clinical characteristics, primary treatments, survival, and patient-reported outcomes for prostate cancer in their jurisdictions. Data linkages include state-based hospital patient records and central cancer registries, with additional linkages to national data on prescribed medicines, procedures, and deaths; refer Figure 1.

Authorisation by contributing custodians and ethics committees, and funding from the Movember charity, has enabled the more detailed evaluation of patient follow-up after the initial treatment to examine historical trends, health service utilisation, disparities and gaps, and long-term patient outcomes.

## Results

South Australian study cohort consists of all men in the SACR (South Australia's central cancer registry) who were diagnosed with prostate cancer [ICD10=C61] as well as any additional men recorded in the PCOR-SA diagnosed from January 2002 to June 2021 (n=25,000). Men who do not have SA addresses recorded in either source are excluded from the South Australian pilot. The Tasmanian cohort is younger (established 2015) and smaller (n=2080), but for comparison is linked with the equivalent local and national datasets.

The South Australian data linkage is being undertaken by SA NT DataLink, with the Tasmanian linkage undertaken by the Tasmania Data linkage Unit. For both jurisdictions, the national datasets are being linked by the Australian Institute for Health and Welfare (AIHW).

## Conclusion

Since the Australian health system involves components managed at both the State and Federal levels, multijurisdictional data linkage adds additional complexity to linkage projects. Undertaking this project was not without its challenges, but it demonstrates what can be achieved and the value of enhancing high-quality clinical registry data to address important outcomes in prostate cancer.

